# Nonvolatile and ultra-low-loss reconfigurable mode (De)multiplexer/switch using triple-waveguide coupler with Ge_2_Sb_2_Se_4_Te_1_ phase change material

**DOI:** 10.1038/s41598-018-34419-2

**Published:** 2018-10-29

**Authors:** Weifeng Jiang

**Affiliations:** College of Electronic and Optical Engineering, Nanjing University of Posts and Telecommunications, Nanjing, 210023 China

## Abstract

Mode-division multiplexing (MDM) is a promising approach to dramatically enhance the transmission capacity. A reconfigurable mode (De)multiplexer/switch (RMDS) is a key component for the flexible mode routing in the MDM network. A nonvolatile and ultra-low-loss RMDS is proposed via a triple-silicon-waveguide directional coupler with the Ge_2_Sb_2_Se_4_Te_1_ (GSST) phase change material (PCM). The nonvolatile property of GSST makes it attractive to reduce the switching power-consumption. Benefiting from the low loss of the GSST-PCM at both amorphous and crystalline states, an RMDS with an ultra-low loss and a high extinction-ratio can be realized. The proposed RMDS is optimally designed by using the full-vectorial finite element method and 3D full-vectorial finite difference time domain method. The numerically simulated results show that a compact RMDS is with the extinction ratios of 18.98 dB and 22.18 dB, ultra-low insertion losses of 0.10 dB and 0.68 dB for the “OFF” and “ON” states, respectively at the operating wavelength of 1550 nm. An ultra-wide bandwidth of 100 nm is achieved for both the “OFF” and “ON” states.

## Introduction

Mode-division multiplexing (MDM) has been attracted much attention as a promising approach for dramatically enhancing the transmission capacity^1,2^. MDM offers a new dimension for the optical network by exploiting orthogonal spatial modes as multiple signal channels for each operating wavelength, which can further significantly increase the spectral efficiency. To build a MDM network on-chip, many key components have been reported, including the mode multiplexers (MUXs)/demultiplexers (DeMUXs)^3^, mode filters^4^, mode converters^5^, two-mode power splitter^6^, mode switches^7^ and so on. Among them, the reconfigurable mode (De)MUX/switch (RMDS) is a basic and indispensable block for a reconfigurable and flexible MDM network, which can switch and route data signals in the multimode channels. However, the RMDS for a MDM network is more complex and requires new mechanisms to achieve the mode switchable functionality, compared with the conventional single-mode (SM) optical switches in the wavelength-division multiplexing (WDM) networks^8^.

Only a few RMDSes have been reported based on the Mach–Zehnder interferometers (MZIs)^9^, micro-ring resonators^10^, and multimode interference (MMI) structures with phase shifters^11^. One approach could be the use of the MZIs with phase shifters. A 2 × 2 multimode optical switch was experimentally achieved with a link-crosstalk for all four modes of <−18.8 dB, composed of two mode DeMUXs, n 2 × 2 SM optical switches and two mode MUXs^9^. This reported approach is based on the independent processing of the single modes’ signals using SM switches, in which the input multimode signals are firstly all converted into the fundamental mode (mode-conversion process); and then, the optical signals are switched by processing of individual channels using conventional optical switches (mode-switching process); finally, the SM signals are reconverted into their original higher-order modes at the output ports (mode-reconversion process). Although this approach can provide a mode switch with a high-performance, this structure would also need extra mode-converters and a large footprint. Another approach could be the use of the micro-ring resonators based structures. B. Stern *et al*. have proposed and demonstrated an on-chip MDM switch based on the micro-rings, which routes four data channels with a low crosstalk (CT) of <−16.8 dB^10^. However, this reported approach is also based on the mode-conversion and mode-reconversion mechanism. In addition, the operating bandwidth and thermal stability of the MDM switch are limited by the micro-ring resonators. Another approach could be the use of the combination of the MMI, Y-branches, and phase shifters. A high-speed two-mode switch has been demonstrated by using this approach, which can achieve a measured switching extinction ratio (ER) of 12.5 dB^11^. However, due to the weak nonlinear electro-optic (EO) effect of silicon material, the footprint of the reported mode-switch is as large as 0.3 mm × 0.6 mm. In order to achieve a compact RMDS with the high performance, the light-matter-interaction needs to be improved inside the device by introducing new structures and mechanisms.

Recently, optical phase change materials (PCMs) based SM optical switches have emerged for on-chip signals switching and routing, including indium tin oxide (ITO), graphene, vanadium dioxide (VO_2_) and Ge_2_Sb_2_Te_5_ (GST)^12–15^. The high light-matter-interactions and compact footprints can be achieved benefiting from giant index-change (>1) of these PCMs. However, two mostly used PCMs, VO_2_ and GST, suffer from high optical propagation losses even in their dielectric states. More recently, a new PCM, Ge_2_Sb_2_Se_4_Te_1_ (GSST) has been reported^16,17^, which exhibits significantly low propagation loss at dielectric state compared to the traditional PCMs. An ultra-low extinction coefficient, k = (1.8 ± 1.2 × 10^−4^) of the GSST at dielectric state was measured at the wavelength of 1550 nm^17^. Particularly, a large index-change of the GSST can be generated by the self-holding phase-transition between the amorphous (a-) and crystalline (c-) states, which can enable the nonvolatile capability to sustain the switches’ states even in the absence of external powers. Therefore, a nonvolatile RMDS with a high performance would be achieved by utilizing the GSST-PCM.

In this paper, we propose and optimise a nonvolatile and ultra-low-loss RMDS via a triple-waveguide directional coupler (DC) with the GSST-PCM and a two-waveguide DC. An input quasi-TM_0_ mode can be correspondingly converted to the quasi-TM_1_ modes of two individual multimode waveguides (WGs) according to a- and c-states of the GSST-PCM, respectively. An ultra-compact footprint, ultra-low losses and high ERs of the proposed RMDS would be achieved benefiting from the GSST-PCM and triple-WG DC structure. The proposed RMDS is optimised by using the full-vectorial finite element method (FV-FEM) and 3D full-vectorial finite difference time domain method (3D-FV-FDTD).

## Results

### Schematic and Principle

**Figure 1:**
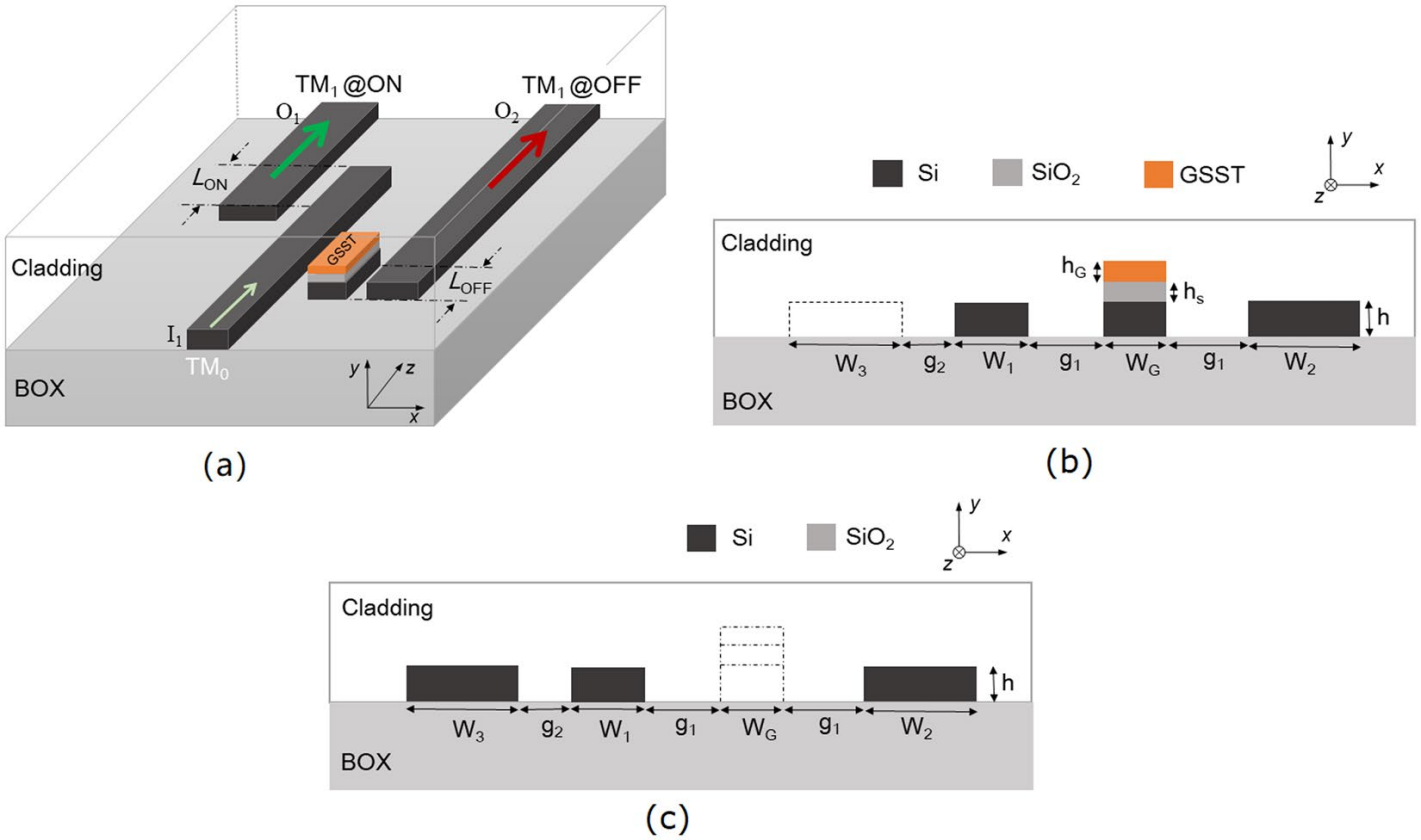
Nonvolatile reconfigurable mode (De)MUX/switch (RMDS). (**a**) Schematic of the proposed RMDS; (**b,c**) are cross-sections of the triple- and two-WG DCs over the lengths of *L*_OFF_ and *L*_ON_, respectively.

The schematic diagram of the proposed RMDS is shown in Fig. 1(a), consisting of a triple-WG DC and a two-WG DC. The triple-WG DC is comprised of an input SM-WG-1, a central WG-G with GSST, and an external bus WG-2. The input fundamental TM mode can be transferred into the bus WG-2 and outputs at port O_2_, when the state of the GSST is amorphous at “OFF” state of the RMDS. While the c-GSST is triggered at “ON” state, the input quasi-TM_0_ mode would propagate along the input SM-WG-1 without mode coupling due to the phase-mismatching. Following this, the input quasi-TM_0_ mode will be converted to the quasi-TM_1_ mode in the bus WG-3 of the two-WG DC and output at port O_1_. It can be observed from Fig. 1(a) that the coupling lengths are denoted by *L*_OFF_ and *L*_ON_ for the triple- and two-WG DCs, respectively. The cross-sections of the triple- and two-WG DCs over the lengths of *L*_OFF_ and *L*_ON_ are shown in Fig. 1(b,c), respectively. The widths of the input SM-WG-1, central WG-G, bus WG-2 and -3 are represented by W_1_, W_G_, W_2_ and W_3_, respectively. The height of the silicon layer of these four WGs is denoted by h, which is chosen to be a common height, h = 220 nm of the silicon WG in this case. It can be observed from Fig. 1(b) that for the triple-WG DC, the separation in between each pair of three WGs is denoted by g_1_. The heights of the GSST and intermedium silica layers of the central WG-G are denoted by h_G_ and h_s_, respectively. It can also be observed that for the two-WG DC, the separation between the input SM-WG-1 and the bus WG-3 is denoted by g_2_. In this case, the refractive indices of the silicon and silicon dioxide are taken as 3.47548 and 1.46, respectively at the operating wavelength of 1550 nm. A silica cladding is used to protect the silicon WGs. The refractive indices of the a- and c-GSST are taken as 3.39 + (1.8 ± 1.2) × 10^−4^i and 5.14 + 0.42i, respectively^17^.

The phase change of the GSST could be supposed to be similar to that of the GST-PCM^18^, which can be triggered via thermo-optic (TO), EO/electro-thermal (ET), and all-optical approaches^19^. In general, the a-GSST can be obtained by heating the c-GSST above the melting point followed by rapid cooling (<1 ns). The c-GSST can be achieved by heating the a-GSST to be crystallized. By introducing a heater above the GSST, the phase state of the GSST can be simply adjusted based on the TO effect. An alternative TO approach could be the use of the pulsed laser to achieve the photo-thermal heating and then change the phase state^20^. For the EO or ET based approach, the silicon sections can be doped as upper and lower contacts and then the applied voltage across the doped silicon can result in the desired thermal phase change via Joule heating current^21^. For the all-optical approach, a pump light can be used to go through the c-GSST section and be partly absorbed by c-GSST due to the relatively high absorption loss of c-GSST, which can also provide the desired thermal phase change for re-amorphization^22^. However, it may be difficult to achieve recrystallization due to the ultra-low extinction coefficient, k = (1.8 ± 1.2 × 10^−4^) of a-GSST. In this case, the phase transition of the GSST is expected to be achieved by using the pulsed laser induced photo-thermal heating, which can be implemented without any electrode. In future, the approach based on the micro-heater or electro-thermal effect may be preferable for flexible photonic integrated circuits.

### Characterization of mode properties

**Figure 2:**
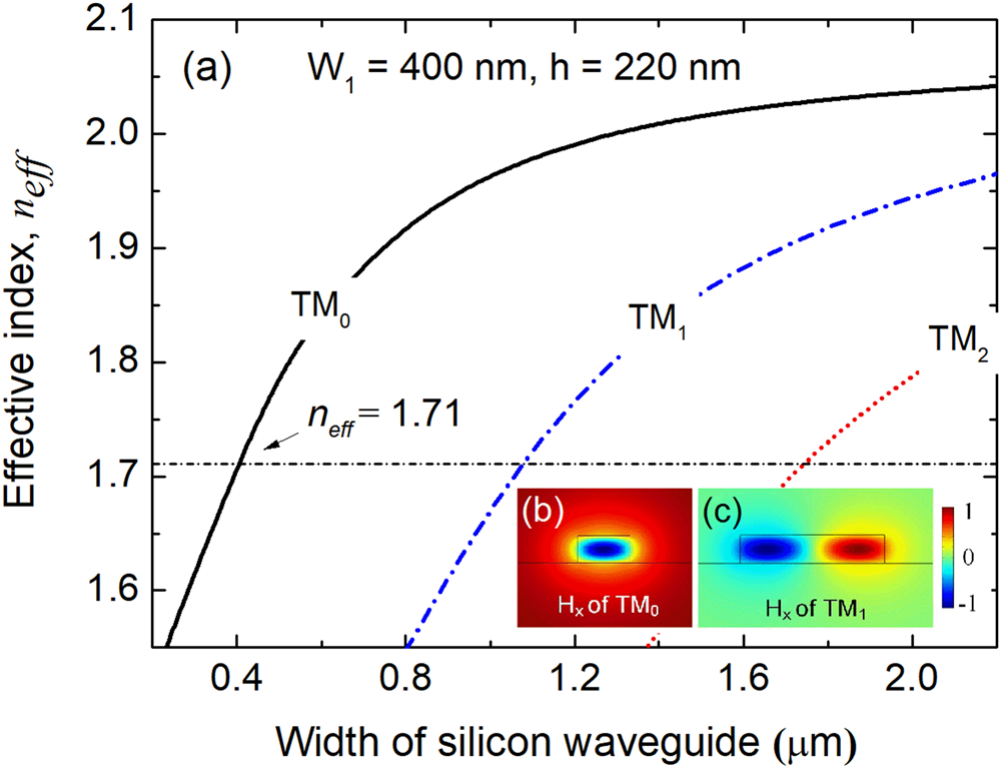
Phase-matching condition for isolated silicon waveguides. (**a**) Variations of the effective index with the width of the silicon waveguide; (**b**,**c**) are the H_x_ mode fields of the phase-matched quasi-TM_0_ and quasi-TM_1_ modes, respectively.

Firstly, the design of the triple-WG DC section with the GSST-PCM is studied by using the FV-FEM. The proposed triple-WG DC operates at two states: (a) for the “OFF” state with a-GSST, the input quasi-TM_0_ mode is converted to the quasi-TM_1_ mode of the bus WG-2 under the phase-matching condition; (b) for the “ON” state with c-GSST, the input quasi-TM_0_ mode propagates along the input SM-WG-1 without any mode coupling. To achieve a maximum mode coupling efficiency at “OFF” state, the phase-matching condition for the input SM-WG-1, central WG-G with a-GSST and the bus WG-2 should be satisfied for the triple-WG DC. Variations of the effective index with the width of the silicon WG are shown in Fig. 2(a), in which the size of the input SM-WG-1 is taken as W_1_ × h = 400 nm × 220 nm. The effective index of the quasi-TM_0_ mode of the input SM-WG-1 is calculated to be n_eff_ = 1.71, denoted by a horizontally dash-dotted line in Fig. 2(a). The phase-matched width of the bus WG-2 is determined to be W_2_ = 1.075 μm. The H_x_ mode fields of the phase-matched quasi-TM_0_ and quasi-TM_1_ modes are shown in Fig. 2(b,c), respectively.

**Figure 3:**
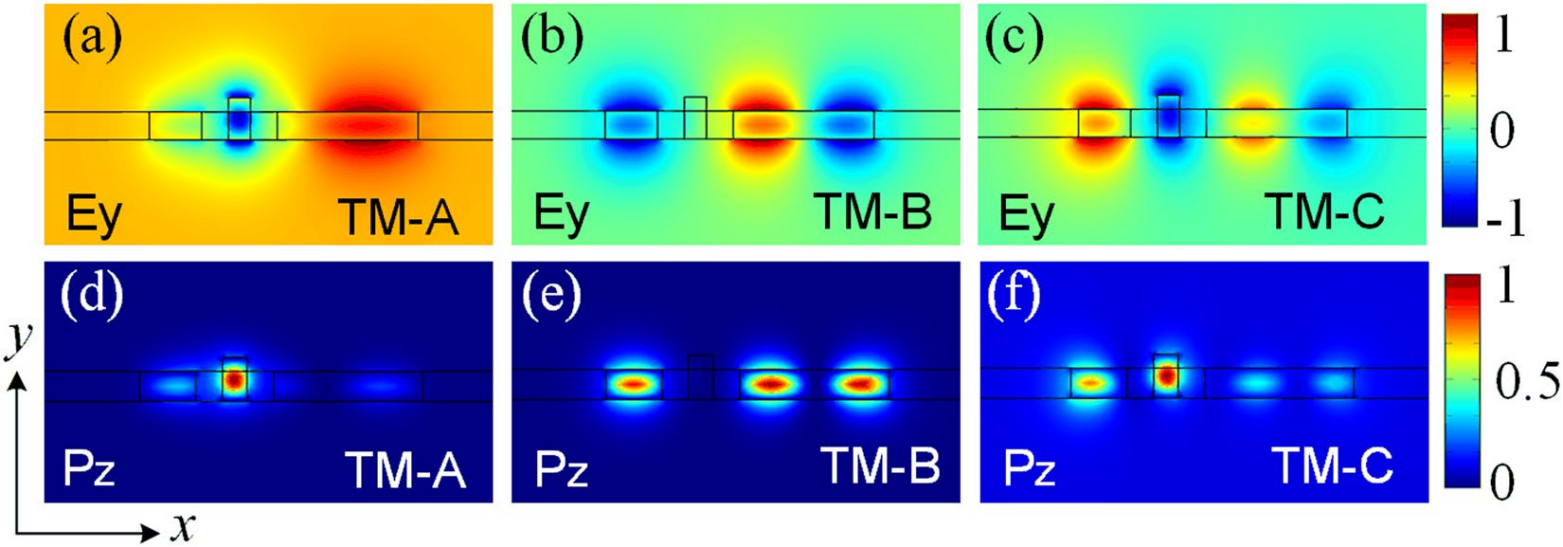
Supermode fields of the triple-waveguide directional coupler. (**a**–**c**) are the E_y_ fields of the TM-A, TM-B and TM-C supermodes, respectively; (**d**–**f**) are the P_z_ (x, y) fields of the TM-A, TM-B and TM-C supermodes, respectively.

For the triple-WG DC, more supermodes would be generated compared to the two-WG DC. In this case, the input quasi-TM_0_ should be phase-matched with the quasi-TM_1_ mode of the bus WG-2. Hence, three supermodes (TM-A, TM-B, and TM-C) are studied and the supermode fields are shown in Fig. 3. In the calculation, the parameters of the triple-WG DC are set as: W_1_ = 400 nm, W_2_ = 1.075 μm, g_1_ = 200 nm, h_G_ = 100 nm and h_s_ = 0 nm. The E_y_ fields of the TM-A, TM-B, and TM-C supermodes are shown in Fig. 3(a–c), respectively. The Poynting vectors, P_z_ (x, y) of these three supermodes are also calculated and shown in Fig. 3(d–f), respectively. To meet the phase-matching condition for this triple-WG DC, the effective indices of the TM-A, TM-B, and TM-C supermodes must satisfy the following condition^23^:

1 $${n}_{A}+{n}_{C}=2{n}_{B}$$

where n_A_, n_B_, and n_C_ are the effective indices of the TM-A, TM-B, and TM-C supermodes, respectively.

**Figure 4:**
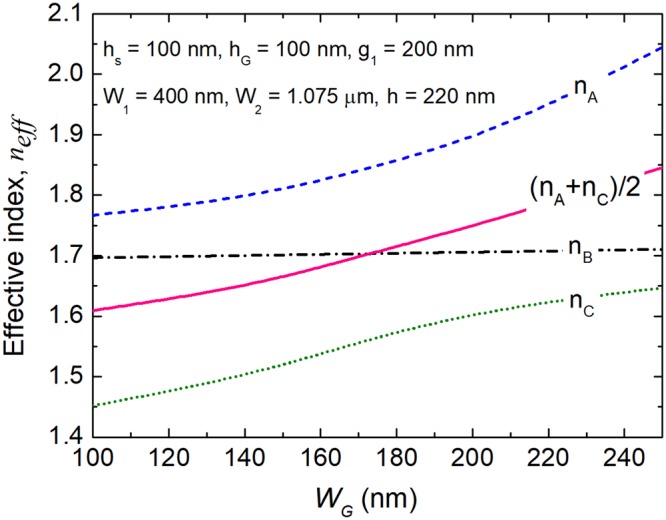
Phase-matching condition for the triple-WG DC. Variations of the effective index with the width of the central WG-G.

Variations of the effective indices of n_A_, n_B_, n_C_, and (n_A_ + n_C_)/2 with the width of the central WG-G are shown in Fig. 4 and denoted by the dashed blue, dash-dotted black, dotted green and solid pink lines, respectively. The triple-WG DC with the parameters of W_1_ = 400 nm, W_2_ = 1.075 μm, h = 220 nm, g_1_ = 200 nm, h_G_ = 100 nm and h_s_ = 100 nm is taken as an example. The effective indices of the n_A_, n_C_ and (n_A_ + n_C_)/2 are increased with the increase of W_G_, while that of the n_B_ is kept constant due to the mode field of the TM-B supermode only confined in both the WG-1 and WG-2. According to Equation (1), the phase-matched W_G_ is chosen to be W_G_ = 173 nm.

**Figure 5:**
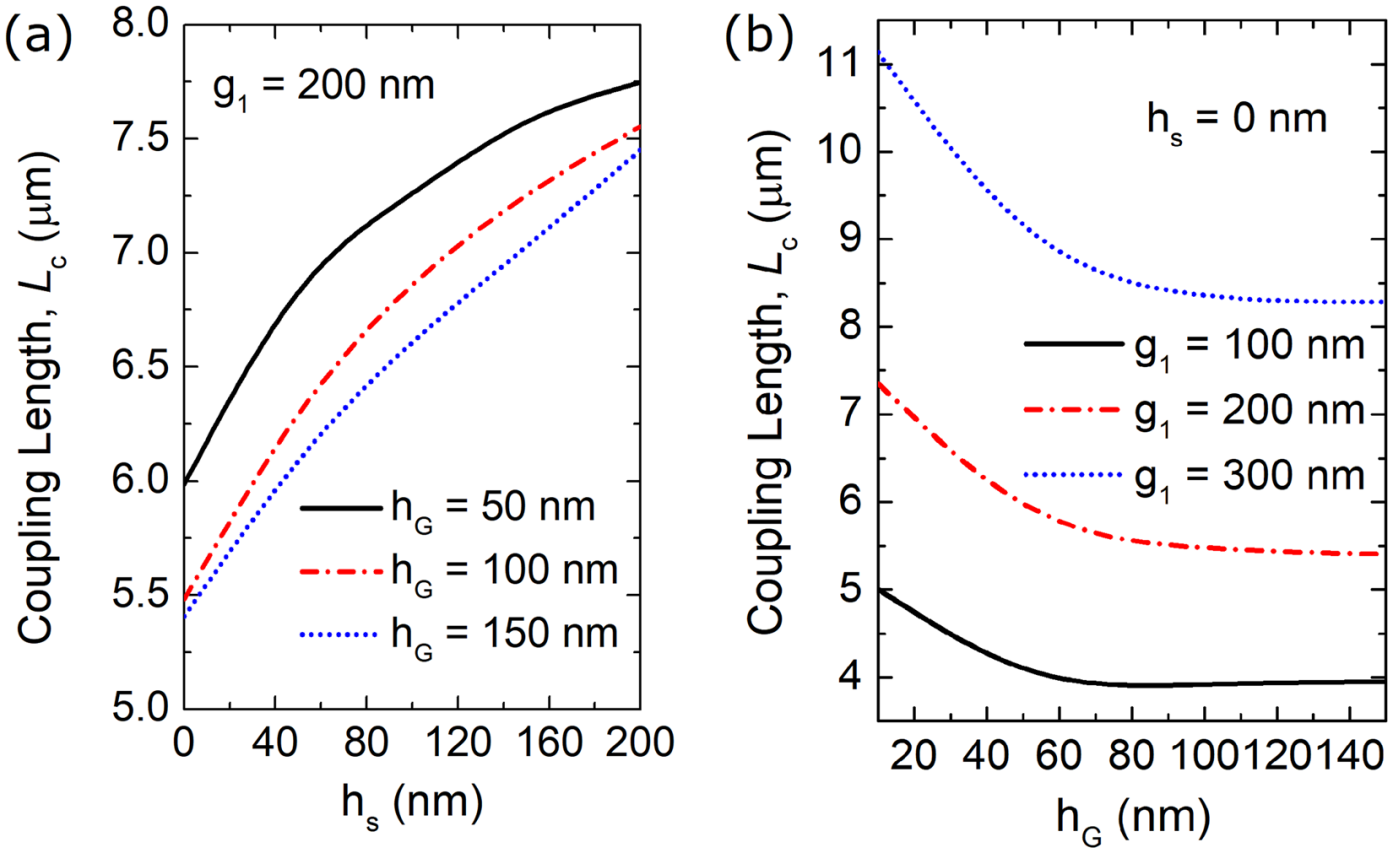
Variations of the coupling lengths with (**a**) height of the silica layer of the central WG and (**b**) height of the a-GSST layer, respectively. In the calculation, W_1_ = 400 nm, W_2_ = 1.075 μm, h = 220 nm; the phase-matched width of the central waveguide, W_G_, should be changed accordingly to meet phase-matching condition.

Next, the coupling length of the triple-WG DC can be calculated by using the formula^23^:

2 $${L}_{C}=\frac{2\pi }{{\beta }_{A}-{\beta }_{C}}=\frac{{{\rm{\lambda }}}_{0}}{2({n}_{A}-{n}_{B})}$$

where *β*_A_ and *β*_C_ are the propagation constants of the TM-A and TM-C supermodes, respectively; λ_0_ is the operating wavelength. In order to achieve a compact RMDS, the coupling length should be optimised to be as short as possible. Variations of the coupling lengths with the heights of the intermedium silica and a-GSST layers are shown in Fig. 5(a,b), respectively. It can be noted from Fig. 5(a) that the coupling lengths for h_G_ = 50 nm, 100 nm and 150 nm are denoted by the solid black, dash-dotted red, and dotted blue lines, respectively. It can be noted from Fig. 5(a) that the coupling lengths are monotonically increased with the increase of h_s_. Therefore, a minimum coupling length, *L*_OFF_ would be obtained in the absence of the intermedium silica layer of the central WG-G. It can also be noted from Fig. 5(a) that a shorter coupling length would be achieved with a larger height of the a-GSST layer. It can be noted from Fig. 5(b) that the coupling lengths for g_1_ = 100 nm, 200 nm and 300 nm are shown by the solid black, dash-dotted red, and dotted blue lines, respectively. A larger separation would induce a longer coupling length due to a weaker mode interaction. For a certain separation, the coupling length is decreased with the increase of the height of the GSST. Particularly, the coupling lengths go to the constants at h_G_ = 50 nm, 70 nm and 90 nm for g_1_ = 100 nm, 200 nm and 300 nm, respectively.

**Figure 6:**
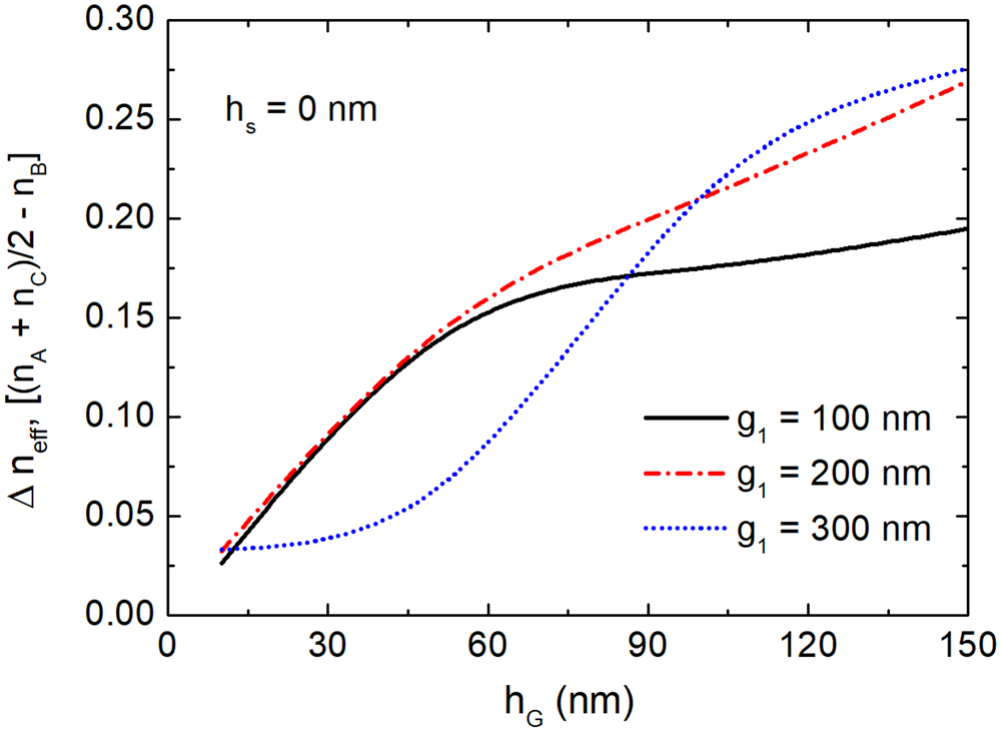
Variations of the difference of the effective index between (n_A_ + n_C_)/2 and n_B_ with the height of the c-GSST layer.

The index difference, Δn_eff_ between (n_A_ + n_C_)/2 and n_B_ at c-GSST state means the intensity of the phase-mismatch of the proposed triple-WG DC at “ON” state, which can determine the mode CT. Variations of the index difference with the height of the c-GSST layer are shown in Fig. 6 and denoted by the solid black, dash-dotted red and dotted blue lines for g_1_ = 100 nm, 200 nm, and 300 nm, respectively. It can be noted that the index differences are increased with the increase of the height of the c-GSST layer, which can be explained by the fact that a larger index-modulation can be generated via a thicker c-GSST layer. If the index difference goes up to 0.15, the heights of the c-GSST layers need to be larger than 57.9 nm, 54.5 nm and 79.8 nm for g_1_ = 100 nm, 200 nm and 300 nm, respectively. The corresponding coupling lengths are calculated to be *L*_OFF_ = 4.0 μm, 5.89 μm and 8.5 μm. Although a more compact coupling length can be obtained with a narrower separation, a higher mode CT would be generated due to the stronger mode interaction. In order to balance the coupling length and the mode CT, the propagation characteristics of the proposed triple-WG DC will be studied by using the 3D-FV-FDTD in the following section.

**Figure 7:**
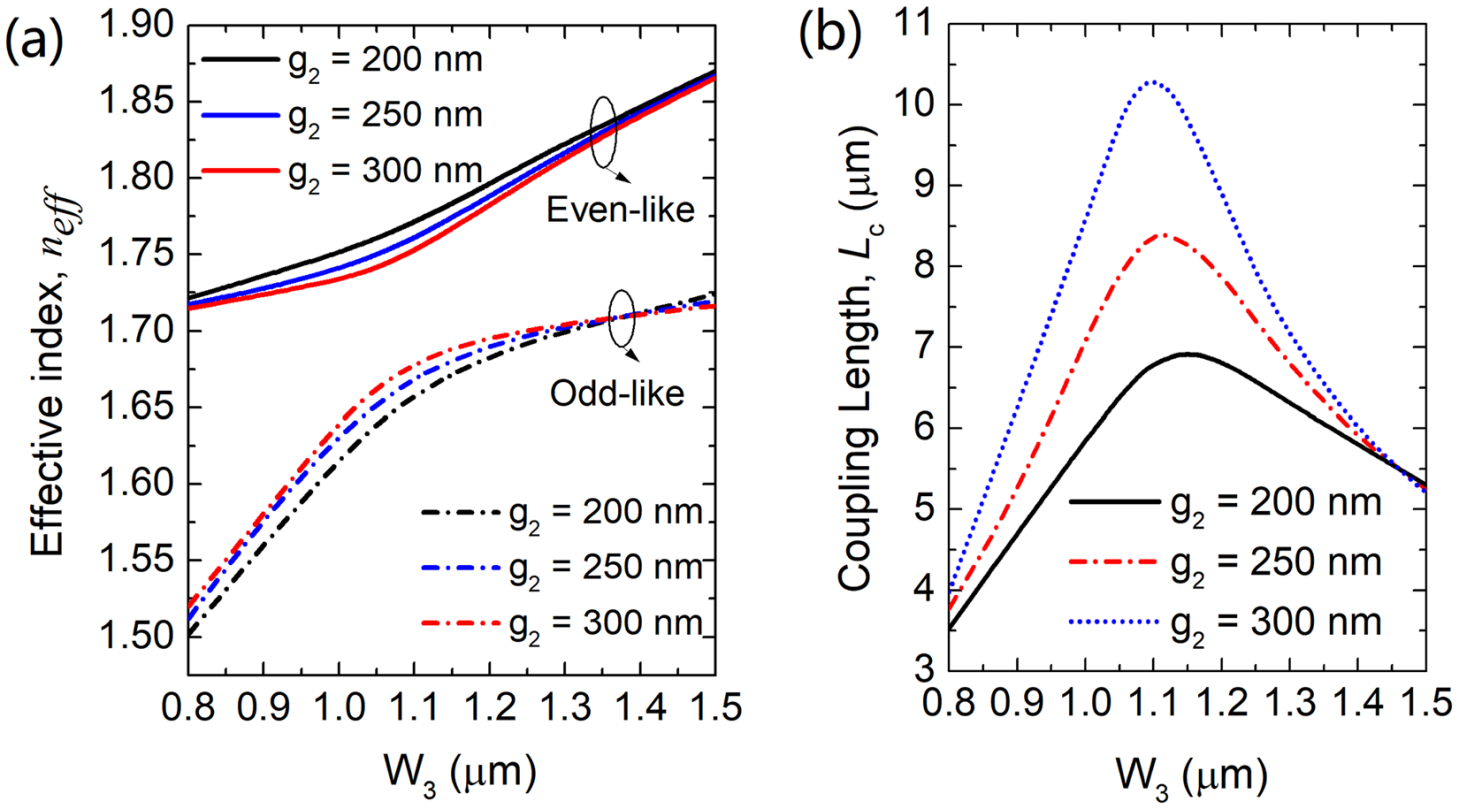
Variations of (**a**) effective index and (**b**) coupling length with the width of the bus WG-3, respectively for the two-WG DC.

**Figure 8:**
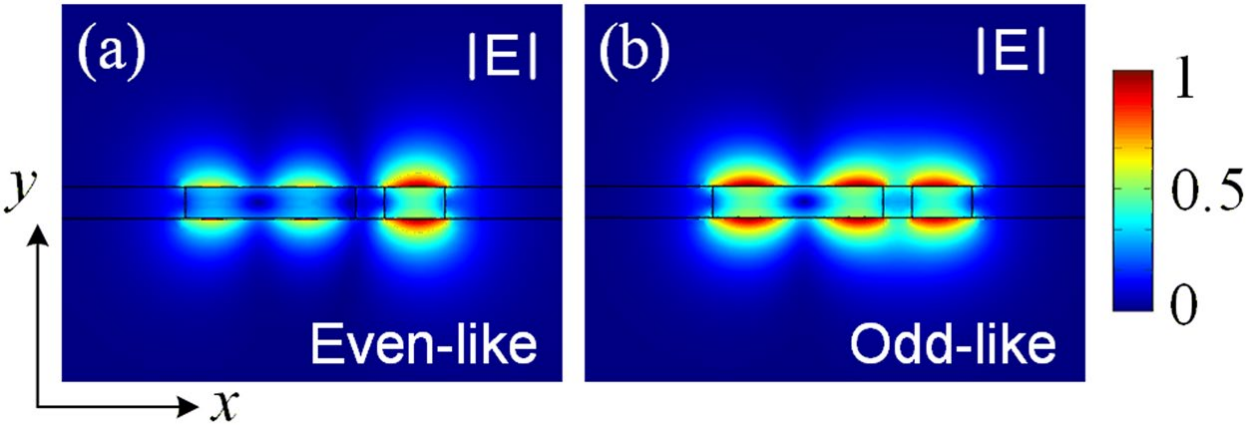
Supermode fields of the two-WG DC. E-field intensities of (**a**) even-like and (**b**) odd-like supermodes, respectively.

For the “ON” state of the proposed RMDS, the input quasi-TM_0_ mode would directly propagate along the input SM-WG-1 and then be coupled to the quasi-TM_1_ mode of the bus WG-3 via a two-WG DC. Although the phase-matching condition between the isolated SM-WG-1 and bus WG-3 can be obtained from Fig. 2, it is essential to study that of the combined two-WG of the DC, which maybe dramatically different with each other due to the strong mode-interaction. Variations of the effective index with the width of the bus WG-3 are shown in Fig. 7(a). The even-like and odd-like supermodes are denoted by the solid and dash-dotted lines, respectively. The separations, g_2_ = 200 nm, 250 nm and 300 nm are shown by the black, blue and red lines, respectively. It can be noted from Fig. 7(a) that the effective indices of the even-like and odd-like supermodes become closer near W_3_ = 1.1 μm and get mixed to form two phase-matched supermodes. The coupling length of the two-WG DC can be calculated based on the formula^24^: *L*_c_ = π/(*β*_even-like_ − *β*_odd-like_), where *β*_even-like_ and *β*_odd-like_ are the propagation constants of the even-like and odd-like supermodes, respectively. Variations of the coupling lengths of the two-WG DC with the width of the bus WG-3 are shown in Fig. 7(b). The coupling length is increased with the increase of the separation. Under the phase-matching conditions (W_3_ = 1.15 μm, 1.11 μm and 1.1 μm), the coupling lengths, *L*_ON_ are calculated to be *L*_ON_ = 6.9 μm, 8.4 μm and 10.3 μm for g_2_ = 200 nm, 250 nm and 300 nm, respectively. The mode fields of two supermodes at g_2_ = 200 nm are calculated by using the FV-FEM and shown in Fig. 8. The E-field intensities of the phase-matched even-like and odd-like supermodes are shown in Fig. 8(a,b), respectively. Similar to the triple-WG DC, a narrower separation of the two-WG DC would induce a higher mode CT. The balance between the coupling length and the mode CT will also be studied by using the 3D-FV-FDTD.

### Operation and bandwidth

**Figure 9:**
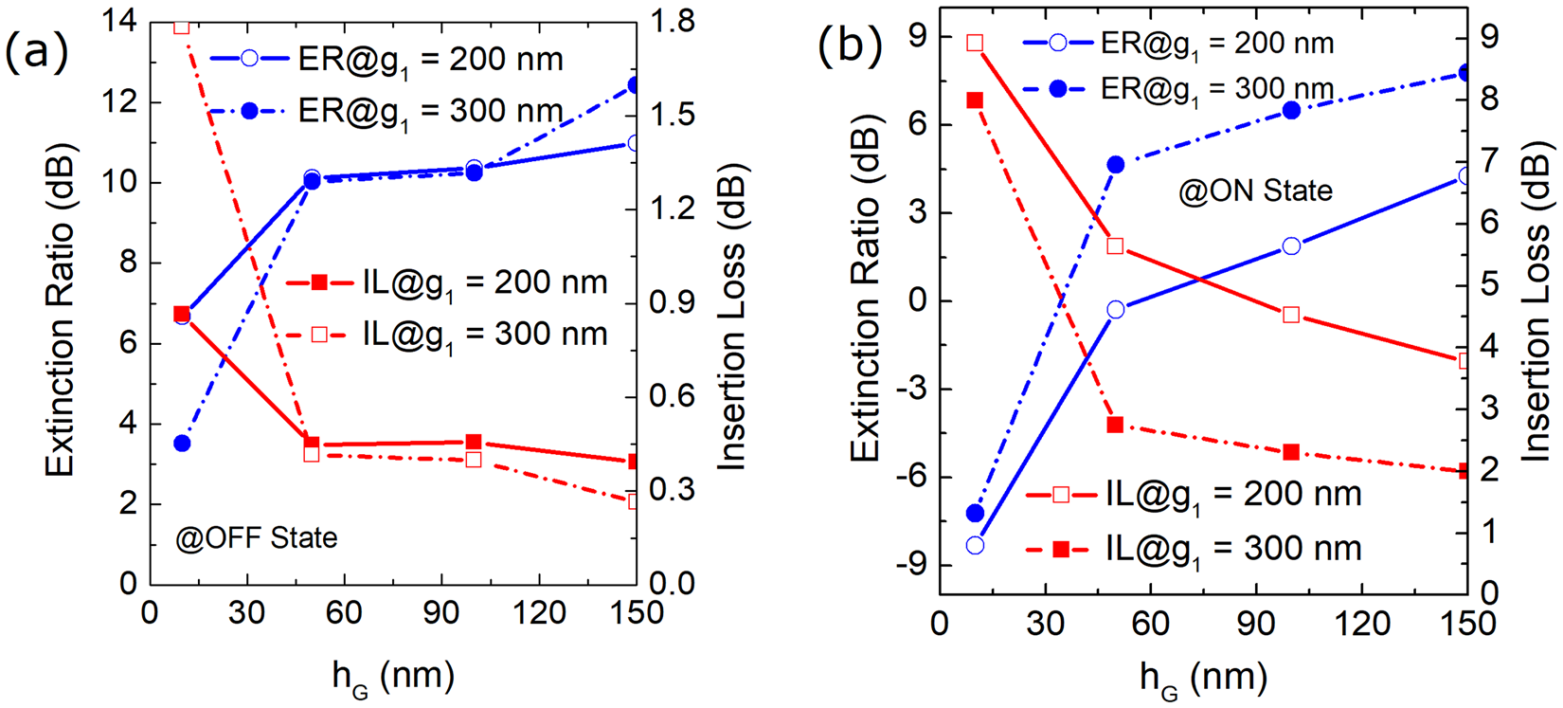
Variations the ER (left y-axis) and IL (right y-axis) with the height of the GSST layer for (**a**) OFF and (**b**) ON states, respectively for the triple-WG DC.

The propagation characteristics of the proposed RMDS are investigated by using the 3D-FV-FDTD. In order to evaluate the performance of the proposed RMDS, the insertion loss (IL) and mode ER are studied and calculated by using the formulas^23^:

3 $$I{L}_{{\rm{OFF}}}=-10{\mathrm{log}}_{10}(\frac{{P}_{{\rm{O2}}}}{{P}_{{\rm{I1}}}})$$

4 $$I{L}_{{\rm{ON}}}=-10{\mathrm{log}}_{10}(\frac{{P}_{{\rm{O1}}}}{{P}_{{\rm{I1}}}})$$

5 $$E{R}_{{\rm{OFF}}}=10{\mathrm{log}}_{10}(\frac{{P}_{{\rm{O2}}}}{{P}_{{\rm{O1}}}})$$

6 $$E{R}_{{\rm{ON}}}=10{\mathrm{log}}_{10}(\frac{{P}_{{\rm{O1}}}}{{P}_{{\rm{O2}}}})$$

where P_I1_ is the input power at port I_1_; P_O1_ and P_O2_ are the output powers at ports O_1_ and O_2_, respectively. The mode CT of the proposed RMDS is calculated by CT = −(IL + ER). For the triple-WG DC section, variations of the ER (left y-axis) and IL (right y-axis) with the height of the GSST layer are calculated based on the 3D-FV-FDTD and shown in Fig. 9(a,b) for the “OFF” and “ON” states, respectively. It can be noted from Fig. 9(a) that at “OFF” state, the ER is increased with the increase of h_G_ for both g_1_ = 200 and 300 nm, while the IL is decreased. Similarly, it can be noted from Fig. 9(b) that the ER is increased and the IL is decreased with the increase of h_G_ for the “ON” state. Although a higher ER and a lower IL can be obtained with a larger h_G_, it would induce a critical fabrication process for the central WG due to the large height-to-width ratio (H/W ratio). In this case, the height of the GSST layer of h_G_ = 150 nm is considered.

**Figure 10:**
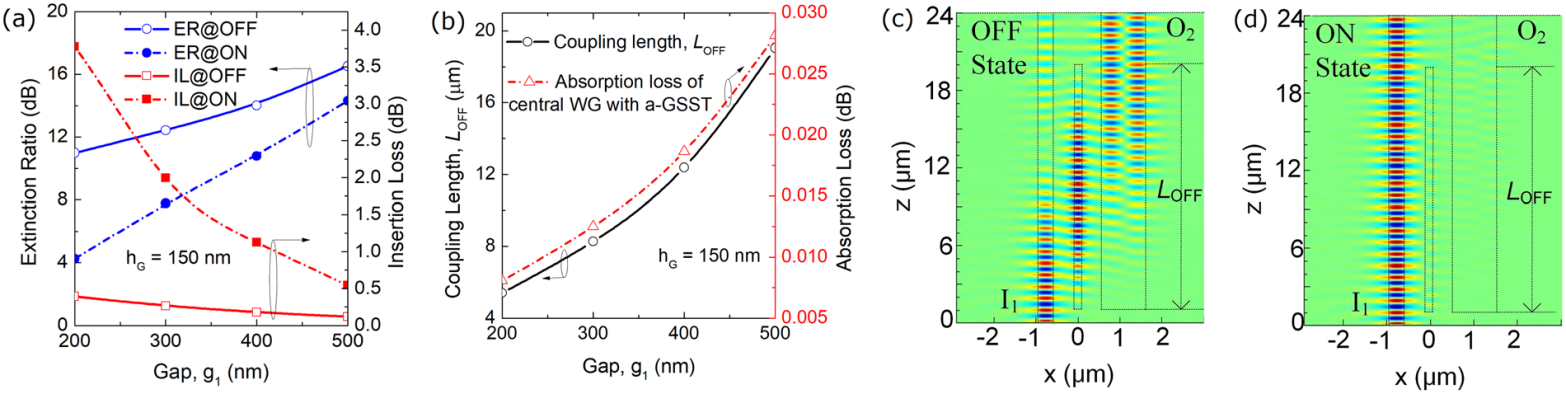
(**a**) Variations the ER (left y-axis) and IL (right y-axis) with the gap, g_1_; (**b**) Variations of the coupling length (left y-axis) and absorption loss of central WG with amorphous-GSST (right y-axis) as a function of the gap, g_1_; E_y_ fields along the propagation direction at (**c**) OFF and (**d**) ON states, respectively for the triple-WG DC.

Variations of the ER (left y-axis) and IL (right y-axis) with the gap, g_1_ are shown in Fig. 10(a). At both “OFF” and “ON” states, the ERs are monotonously increased as a function of the gap, g_1_. At “ON” state, the IL is dramatically decreased from 3.7 dB at g_1_ = 200 nm to only 0.54 dB at g_1_ = 500 nm. The IL at “OFF” state is < 0.4 dB in the whole range and goes to only 0.12 dB at g_1_ = 500 nm, benefiting from the low-loss of the a-GSST-PCM. However, the absorption loss of the a-GSST [k = (1.8 ± 1.2) × 10^−4^] should be taken into account, which is calculated by using the formula^25^: *L*_*abs*_ = 20log_10_[exp(−*β*_im_*L*_OFF_)], where *β*_*im*_ is the imaginary part of the propagation constant of the central WG-G. Variations of the coupling length (left y-axis) and absorption loss of central WG-G with a-GSST (right y-axis) as a function of the gap, g_1_ is shown in Fig. 10(b). It can be noted that with the gap, g_1_ changing from 200 nm to 500 nm, the coupling length, *L*_OFF_ is increased from 5.4 μm to 19.0 μm and then the absorption loss is slightly increased from 0.008 dB (2.1% of IL at “OFF” state) to 0.028 dB (23% of IL at “OFF” state), which is relatively and reasonably small compared to the total IL. Hence, the IL is decreased with the increase of the gap, g_1_, as stated in Fig. 10(a). In this case, an appropriate gap, g_1_ = 500 nm is chosen to balance the ER/IL and *L*_OFF_. The coupling length of the triple-WG DC is calculated to be *L*_OFF_ = 19.0 μm. The E_y_ fields along the propagation direction at “OFF” and “ON” states are shown in Fig. 10(c,d), respectively. It can be observed that at “OFF” state, the input optical power of the quasi-TM_0_ mode is transferred to that of the quasi-TM_1_ mode of the bus WG-2, while the input power propagates along the input SM-WG-1 without mode coupling at “ON” state.

**Figure 11:**
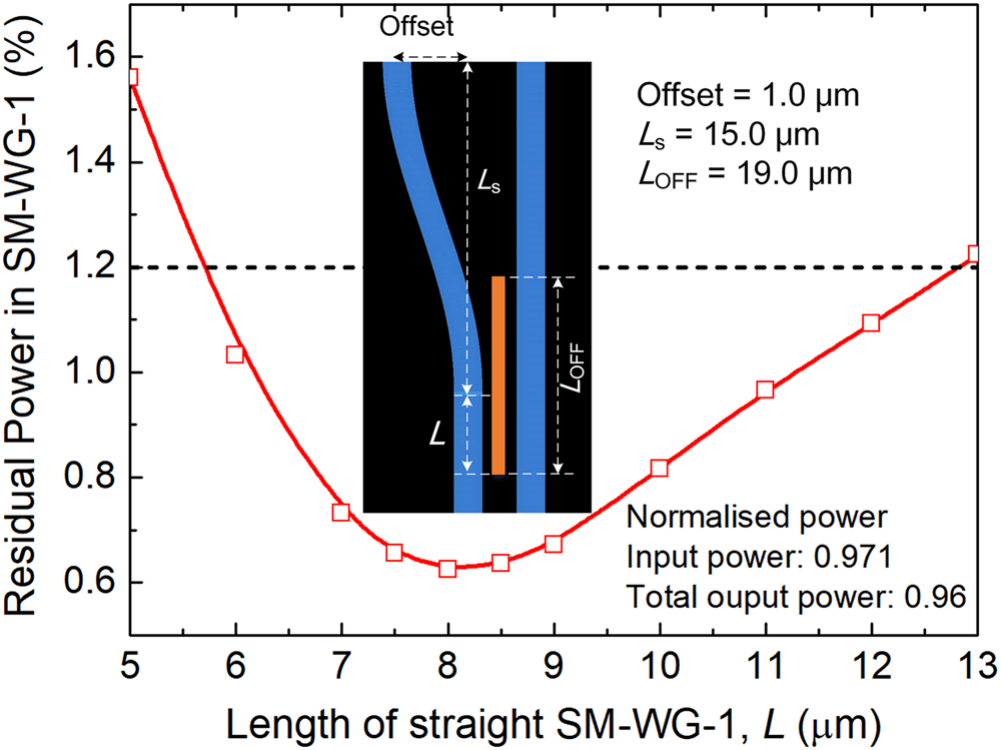
Variations of the normalised residual power in SM-WG-1 with the length of straight section of SM-WG-1, *L*. The inset is the schematic of triple-waveguide coupler with an s-shape bend waveguide.

As shown in Fig. 10(c), there is a week coupling at “OFF” state between the SM-WG-1 and WG-2-O_2_ even if the central WG-G with GSST is cut off. An s-shape bend waveguide could be added to gradually separate the SM-WG-1 and WG-2-O_2_ to reduce unnecessary IL, which is shown in Fig. 11 as an inset. The length, *L*_s_ and offset of the s-shape bend waveguide are chosen to be 15.0 μm and 1.0 μm, which can provide an enough separation between the SM-WG-1 and WG-2-O_2_. We calculated the normalised residual power in SM-WG-1 by varying the length of the straight section of SM-WG-1, *L*. The normalised residual power in SM-WG-1 without an s-shape bend waveguide is calculated to be 1.2%, as denoted by a horizontally dashed-black line in Fig. 11. It can be noted that by implementing an s-shape bend waveguide, the normalised residual power in SM-WG-1 is reduced from 1.2% to only 0.62% with an optimal *L* = 8.0 μm. The IL is slightly decreased from 0.1 dB to 0.078 dB and the mode CT is subsequently decreased from −19.08 dB to −21.9 dB.

**Figure 12:**
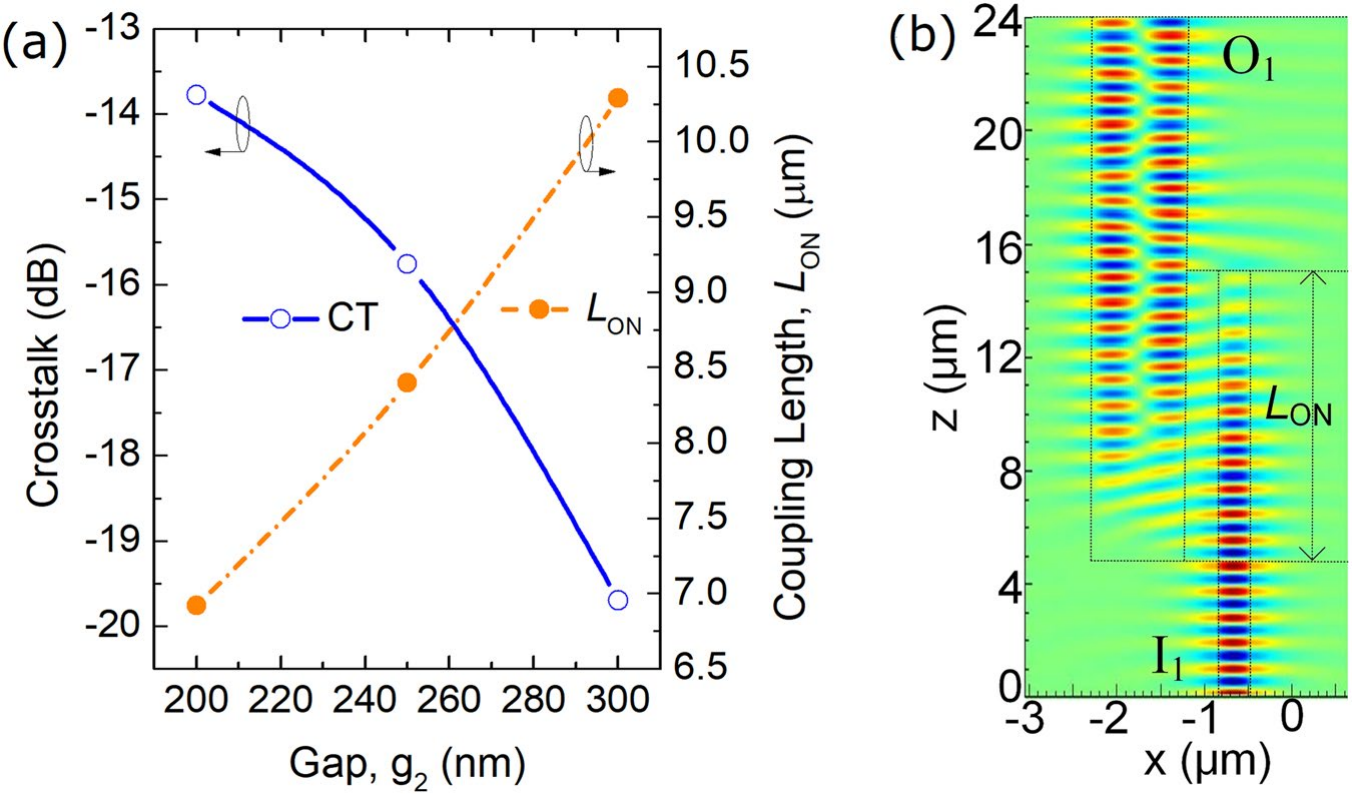
(**a**) Variations of the mode CT (left y-axis) and *L*_ON_ (right y-axis) with the gap, g_2_; (**b**) E_y_ field along the propagation direction for the two-WG DC.

Next, the propagation characteristics of the two-WG DC section are studied by using the 3D-FV-FDTD. Variations of the mode CT (left y-axis) and *L*_ON_ (right y-axis) with the gap, g_2_ are shown in Fig. 12(a). It can be noted that the mode CT is decreased with the increase of the gap, g_2_, while the coupling length is increased. In this case, the gap, g_2_ = 300 nm is selected with an acceptable coupling length of *L*_ON_ = 10.3 μm and a low mode CT of −19.7 dB. The E_y_ field along the propagation direction is shown in Fig. 12(b), which shows that the input quasi-TM_0_ mode is totally converted to the quasi-TM_1_ mode of the bus WG-3.

**Figure 13:**
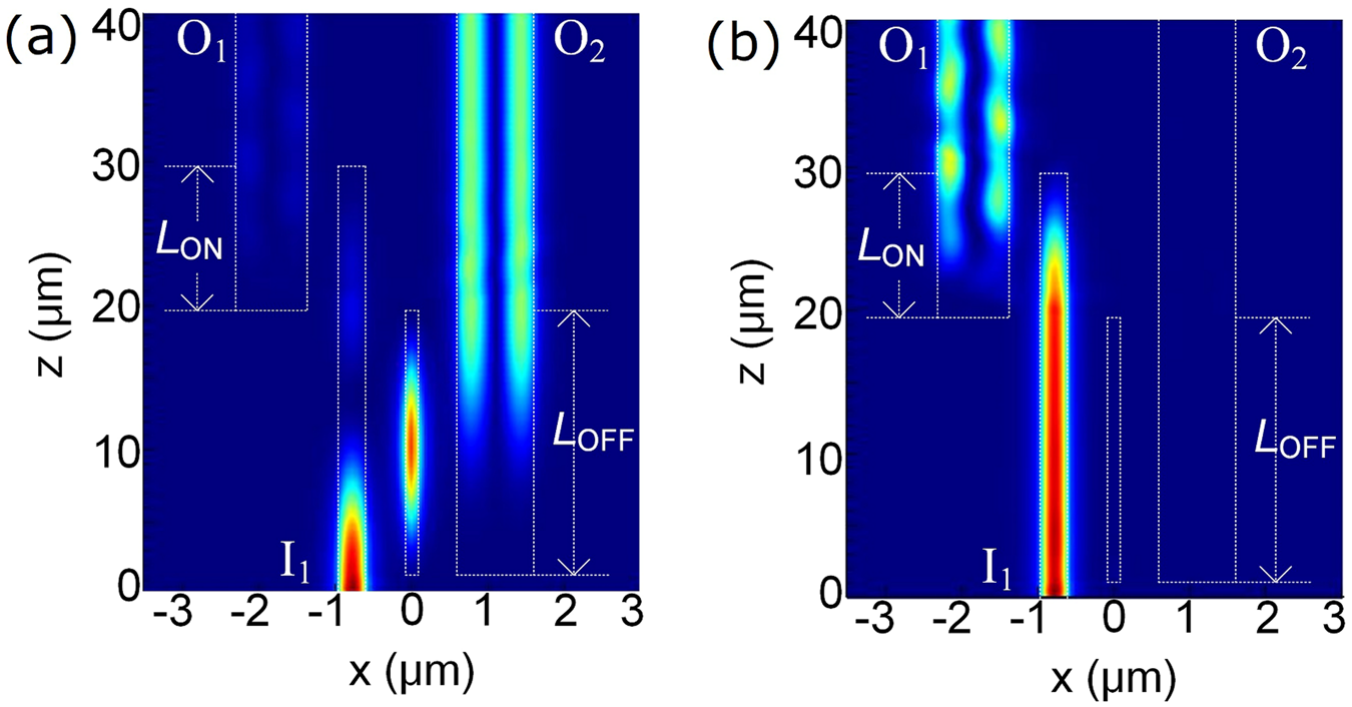
Optical fields along the propagation direction at (**a**) OFF and (**b**) ON states, respectively for the optimised RMDS.

Next, the performance of the optimised RMDS is investigated by utilizing the 3D-FV-FDTD. The optical fields along the propagation direction at “OFF” and “ON” states are shown in Fig. 13(a,b), respectively. At “OFF” state with a-GSST-PCM, the input quasi-TM_0_ mode is switched to the first-order TM-mode of the bus WG-2 and outputs at port O_2_. While at “ON” state with c-GSST-PCM, the input quasi-TM_0_ mode is switched to the quasi-TM_1_ mode of the bus WG-3 and outputs at port O_1_. The ERs (ILs) are calculated to be 18.98 dB (0.10 dB) and 22.28 dB (0.58 dB) for the “OFF” and “ON” states, respectively. The CTs at “OFF” and “ON” states are −19.08 dB and −22.86 dB, respectively. The total coupling length of the optimised RMDS is *L*_OFF_ + *L*_ON_ = 29.3 μm, which is an ultra-compact size compared with that of the MZIs based ones (~several hundreds of micrometers)^9^. We need to pay attention to Fig. 13(b) that the output pattern at “ON” state has a certain oscillation, which indicates that the out-coupled field is actually composed of mixed modes. As the bus WG-3 with the optimised width of 1.1 μm, two TM modes (TM_0_ and TM_1_) and three TE modes (TE_0_, TE_1_, and TE_2_) can be supported in this waveguide. The normalised power of the mixed modes in the bus WG-3 is calculated by using the mode expansion method, which shows that the out-coupled field of the bus WG-3 consists of both the TM_0_ and TM_1_ modes with 2.155% and 97.845% of total output power, respectively. The IL and mode ER at “ON” state would be slightly deteriorated to be 0.68 dB and 22.18 dB, respectively.

**Figure 14:**
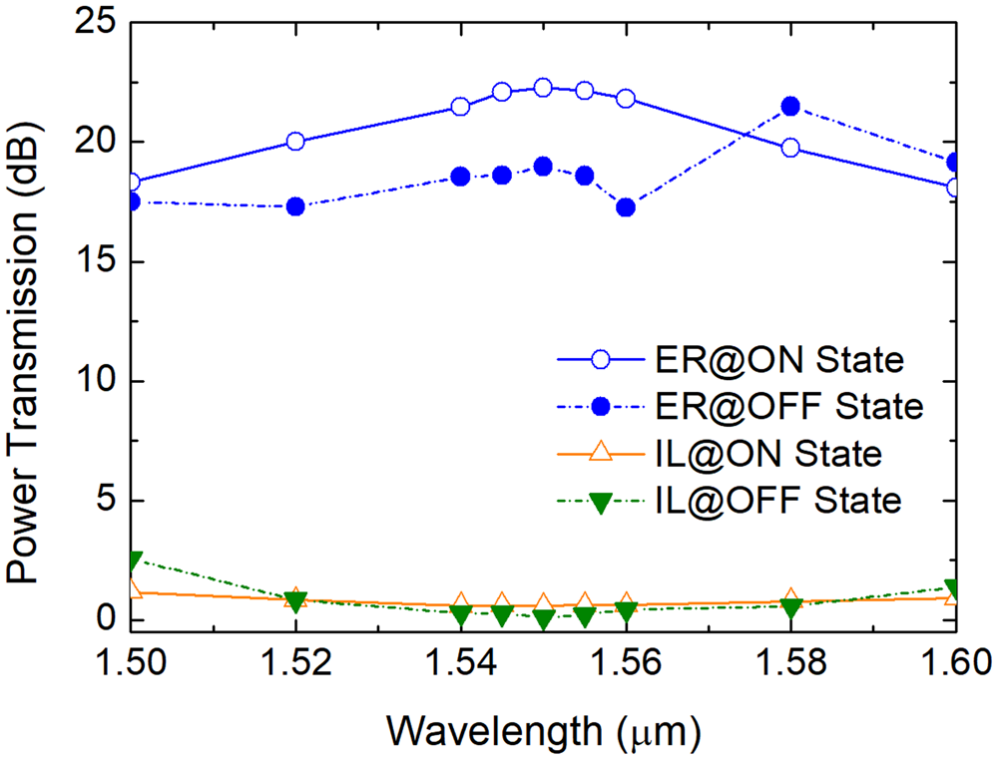
Variations of the power transmission with the operating wavelength of the optimised RMDS.

The bandwidth of the RMDS is key to the on-chip MDM systems for constructing hybrid MDM-WDM networks. Variations of the power transmission with the operating wavelength of the optimised RMDS is shown in Fig. 14. It can be noted that the high ER of >17.3 dB can be achieved for both the states over 100 nm spectral bandwidth. The IL_ON_ is lower than 1.0 dB over 80 nm from 1520 nm to 1600 nm and the 1dB-IL_OFF_ bandwidth is over 60 nm from 1520 nm to 1580 nm. Compared to the MZIs based RMDS, the IL is significantly decreased from several dB to less than 1.0 dB^9^. Compared to the micro-rings based RMDS, the bandwidth is dramatically increased from a narrow bandwidth of only < 13 GHz to 100 nm^10^. The proposed RMDS shows a high performance for providing an efficient approach to construct a hybrid MDM-WDM system.

**Figure 15:**
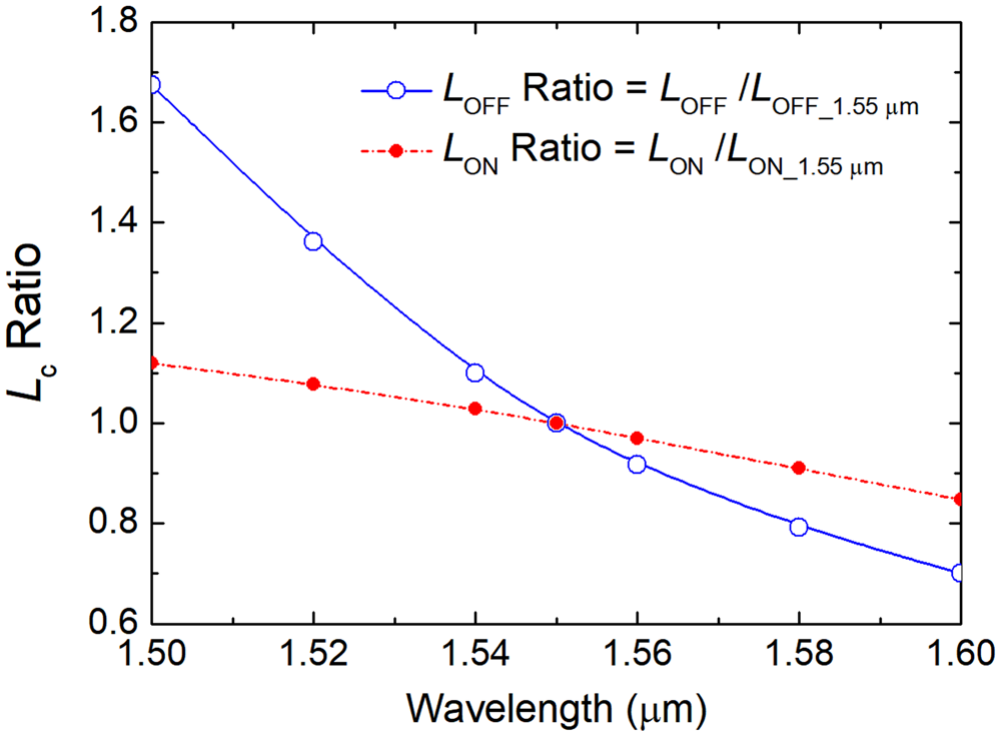
Variations of the ratio of coupling length, [*L*_c_/(*L*_c_ at 1550 nm)) with the operating wavelength.

As shown in Fig. 14, the ER at “OFF” state as a function of wavelength has a large fluctuation. The reason for this fluctuation at “OFF” state can be explained as two aspects: variations of the coupling length and the normalised power of mixed modes in bus WG-3 with the operating wavelength. In order to analyze the change of the coupling length with the operating wavelength, we define the ratio of the coupling length as: *L*_c_ ratio = *L*_c_/(*L*_c_ at 1550 nm). With the optimal parameters of W_1_ = 400 nm, W_2_ = 1.075 μm, W_3_ = 1.1 μm, W_p_ = 134.7 nm, g_1_ = 500 nm, and g_2_ = 300 nm, variations of the ratio of the coupling length for both *L*_OFF_ and *L*_ON_ with the wavelength are shown in Fig. 15. It can be noted that both the *L*_OFF_ ratio and *L*_ON_ ratio are monotonously decreased with the increase of the operating wavelength in between 1500 nm and 1600 nm.

For the triple-WG DC over the bandwidth of 1500 nm ~ 1550 nm, the calculated coupling length would be larger than the physical length, *L*_OFF_ at 1550 nm of the central WG-G, which would induce a residual power remaining in the input SM-WG-1. In addition, a longer coupling length will be achieved for a smaller wavelength. Hence, for the bandwidth in between 1500 nm and 1550 nm, a larger deterioration of the mode ER was induced with a smaller wavelength, as shown in Fig. 14. For the triple-WG DC over the bandwidth of 1550 nm ~ 1600 nm, the calculated coupling length will be shorter than the physical length of the central WG-G. The input quasi-TM_0_ mode will be firstly coupled to the quasi-TM_1_ mode in the bus WG-2 and then be coupled back to the central WG-G, and then may be coupled back to the input SM-WG-1 depending on the calculated coupling length, which may also induce a residual power remaining in the input SM-WG-1. However, the mode ER at 1580 nm is larger than that at 1550 nm, as shown in Fig. 14. We checked the propagation field of the triple-WG DC at 1580 nm and found that the back-coupled field in the central WG-G radiates at the end of this waveguide and with only a tiny power (0.62%) coupled back to the input SM-WG-1. But, 1.7% and 0.884% of the total power is coupled back to the input SM-WG-1 for 1560 nm and 1600 nm, respectively. Therefore, a fluctuation is induced in between 1550 nm and 1600 nm, as shown in Fig. 14.

**Figure 16:**
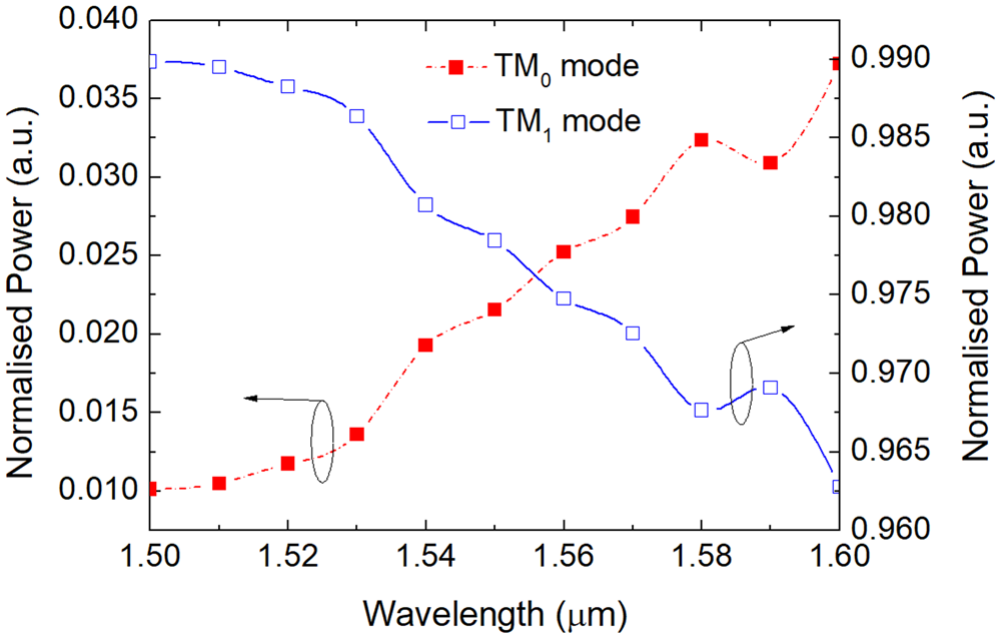
Variations of the normalised power of mixed mode in bus WG-3 versus wavelength.

Next, variations of the normalised power of mixed modes in the bus WG-3 with the operating wavelength are calculated and shown in Fig. 16. It can be noted that for the two-WG DC, the power out-coupled to the quasi-TM_0_ mode of the bus WG-3 is increased with the increase of the operating wavelength, whereas that out-coupled to the quasi-TM_1_ mode is decreased. We should pay attention to the wavelength in between 1580 nm and 1600 nm, where a fluctuation of the normalised power of mixed modes is existed, subsequently further enlarges the variation of the mode ER at “OFF” state shown in Fig. 14.

## Conclusion

In conclusion, we have proposed and optimised a nonvolatile and ultra-low-loss RMDS, consisting of a triple-WG DC with the GSST-PCM and a two-WG DC. Due to the self-holding phase-transition between a- and c-states of the GSST-PCM, the nonvolatile capability to sustain the switches’ states can be achieved to reduce the power-consumption. The proposed RMDS has been optimally designed by using the FV-FEM and 3D-FV-FDTD. The ultra-low insertion-losses of 0.10 dB and 0.68 dB of the optimised RMDS have been achieved for the “OFF” and “ON” states, respectively, benefiting from the low losses of the GSST-PCM at both a- and c-states. The mode CTs at “OFF” and “ON” states were −19.08 dB and −22.86 dB, respectively. The proposed RMDS was with a compact coupling length of 29.3 μm and high ERs of 18.98 dB and 22.18 dB for the “OFF” and “ON” states, respectively. A reasonable high ER of >17.3 dB has been achieved for both the states over 100 nm bandwidth, which offers the potential application in the S + C + L band MDM-WDM networks. The proposed RMDS can be applied in the MDM networks to provide a flexible mode routing and switching.

## Methods

The mode characteristics of the isolated silicon waveguides and the combined triple-waveguide directional coupler (DC) are calculated by using the full-vectorial finite element method (FV-FEM). The refractive indices of the silicon, silicon dioxide, amorphous- and crystalline-Ge_2_Sb_2_Se_4_Te_1_ (GSST) are taken as 3.47548, 1.46, 3.39 + (1.8 ± 1.2) × 10^−4^i and 5.14 + 0.42i, respectively at the operating wavelength of 1550 nm. The phase-matching conditions for two-waveguide and triple-waveguide based DCs are determined by using the FV-FEM. The coupling lengths and the difference of the effective index are also optimized by using the FV-FEM. The propagation characteristics (propagation fields, mode crosstalk, insertion loss, and mode extinction ratio) of the optimised reconfigurable mode (De)MUX/switch (RMDS) are studied by using the 3D full-vectorial finite difference time domain (3D-FV-FDTD) method. The bandwidth of the optimised RMDS is also evaluated by using the 3D-FV-FDTD. The normalised power of the mixed mode in the bus waveguide-3 is investigated by using the mode expansion method (MEM).
